# Comparison of the cytokine responses to acute strength exercise between oral contraceptive users and naturally cycling women

**DOI:** 10.1007/s00421-023-05275-4

**Published:** 2023-07-15

**Authors:** Hannah L. Notbohm, Lisa Umlauff, Wilhelm Bloch, Moritz Schumann

**Affiliations:** 1 Department of Molecular and Cellular Sports Medicine, German Sport University, Am Sportpark Müngersdorf 6, 50933 Cologne, Germany; 2 Division of Training and Movement Science, University of Potsdam, Potsdam, Germany

**Keywords:** Interleukin-1, Interleukin-8, Follicular phase, Menstrual cycle, Hormonal contraception

## Abstract

**Purpose:**

Cytokines are released as part of an inflammatory reaction in response to strength exercise to initiate muscle repair and morphological adaptations. Whether hormonal fluctuations induced by the menstrual cycle or oral contraceptives affect inflammatory responses to strength exercise remains unknown. Therefore, we aimed to compare the response of cytokines after acute strength exercise in naturally menstruating women and oral contraceptive users.

**Methods:**

Naturally menstruating women (MC, *n* = 13, 24 ± 4 years, weekly strength training: 4.3 ± 1.7 h) and women using a monophasic combined pill (> 9 months) (OC, *n* = 8, 22 ± 3 years, weekly strength training: 4.5 ± 1.9 h) were recruited. A one-repetition-maximum (1RM) test and strength exercise in the squat (4 × 10 repetitions, 70%1RM) was performed in the early follicular phase or pill free interval. Concentrations of oestradiol, IL-1β, IL-1ra, IL-6, IL-8, and IL-10 were assessed before (pre), directly after (post) and 24 h after (post_24_) strength exercise.

**Results:**

IL-1ra increased from pre to post (+ 51.1 ± 59.4%, *p* = 0.189) and statistically decreased from post to post_24_ (– 20.5 ± 13.5%, *p* = 0.011) only in OC. Additionally, IL-1β statistically decreased from post to post_24_ (– 39.6 ± 23.0%, *p* = 0.044) only in OC. There was an interaction effect for IL-1β (*p* = 0.038) and concentrations were statistically decreased at post_24_ in OC compared to MC (*p* = 0.05). IL-8 increased across both groups from post to post_24_ (+ 66.6 ± 96.3%, *p* = 0.004).

**Conclusion:**

We showed a differential regulation of IL-1β and IL-1ra between OC users in the pill-free interval and naturally cycling women 24 h after strength exercise, while there was no effect on other cytokines. Whether this is associated with previously shown compromised morphological adaptations remains to be investigated.

## Introduction

To achieve long-term training adaptations to strength exercise, repair of muscle damage, and restructuring of muscle tissue after acute exercise is essential (Damas et al. 2018). These processes are tightly regulated by a controlled and coordinated inflammatory response, of which cytokines are one of the main mediators (Freidenreich and Volek 2012; Pedersen and Hoffman-Goetz 2000). Generally, from previous research it is known that a local inflammatory response is generated immediately after muscle loading occurs and pro-inflammatory cytokines, such as interleukin 1 beta (IL-1β), interleukin 1 alpha (IL-1α), IL-6 and tumour necrosis factor alpha (TNF-α) are released to initiate muscle remodulation processes (Peake et al. 2015). However, to avoid an excessive pro-inflammatory response, anti-inflammatory cytokines such as interleukin 1 receptor antagonist (IL-1ra), IL-10, IL-4 and tumour growth factor beta (TGF-β) are released further on in the repair process (Petersen and Pedersen 2005).

While the general inflammatory response seems to be well characterised in men (Ihalainen et al. 2014; Izquierdo et al. 2009; Suzuki et al. 2020), less is known on the extent of which hormonal fluctuations in women may impact these responses, especially following acute strength exercise. Inflammatory responses in women distinctly vary to men; for example, evidence suggests that women produce higher levels of antibodies and a larger secretion of inflammatory mediators to external pathogens (Klein and Flanagan 2016). Likely this is at least in part due to differences in sex hormones, with women experiencing regular fluctuations of the sex hormones oestrogen and progesterone throughout the menstrual cycle. The receptors of these hormones are expressed on a variety of immune cells, therefore, indicating that oestrogen and progesterone also play a role in regulating inflammatory responses and the secretion of cytokines from these cells (Kovats 2015; Straub 2007). In the early follicular phase, low oestrogen concentrations and progesterone concentrations may provoke a pro-inflammatory environment, as these have been shown to induce T helper type 1 (Th1) responses and the release of cytokines, such as IL-1β and TNF-α (Salem 2004; Whitcomb et al. 2014). In contrast, high oestrogen concentrations, as experienced during the late follicular phase and luteal phase, have been discussed to have a protective effect on inflammatory responses and lead to a stronger T helper type 2 (Th2) response (Klein and Flanagan 2016; Straub 2007). Therefore, it is likely that hormonal fluctuations may influence the inflammatory response to strength exercise.

The use of oral contraceptives further adds complication to understanding the influence of hormonal fluctuations in women on the inflammatory responses to strength exercise. Women using oral contraceptives do not experience naturally cycling hormonal concentrations, as circulating hormone concentrations are determined by exogenous hormones. In the active pill phase, endogenous oestrogen and progesterone concentrations are suppressed by the daily dose of exogenous hormones. During the pill-free interval, there is a withdrawal of exogenous oestrogen and progesterone, which lead to slight increase of endogenous oestrogen concentrations throughout this phase, however, concentrations of oestrogen and progesterone remain low (De Leo et al. 2016). Independent of performing strength exercise, studies have shown oral contraceptives to have an effect on the resting inflammatory status of women. For example, an increased concentration of IL-6 and TNF-α was found in the pill-free interval of young women using oral contraceptives compared to naturally cycling women (Eagan et al. 2021; Hinton et al. 2006), indicating a chronic effect of oral contraceptive use beyond the acute daily dose of exogenous hormones. Furthermore, increased concentrations of high-sensitive C-reactive protein (hs-CRP) and markers of oxidative stress were found in oral contraceptive users, indicating a status of low-grade inflammation in these women (Cauci et al. 2017, 2021; Quinn et al. 2021), which has been speculated to predispose oral contraceptive users to a stronger exercise-induced inflammatory response. In the context of exercise, differences in the inflammatory response after acute cycling exercise (90 min at 65% of peak power) have been shown (Timmons et al. 2005). In this study, women using oral contraceptives showed a reduced IL-6 response in the low hormone phase compared to women in the follicular phase of a natural menstrual cycle. However, research on inflammatory responses in oral contraceptive users and naturally cycling women is limited and results have also been contradictory in some cases, potentially also due to limited control, definition and verification of the cycle phases and oral contraceptives investigated. Furthermore, how the response to acute strength exercise compares, largely remains to be investigated.

As the number of female participants in sports has been rising in recent years and further approximately 50% of female athletes are using oral contraceptives (Martin et al. 2018), better characterising the inflammatory response to strength exercise in women is vital. Therefore, the aim of this study was to analyse a number of cytokines (IL-1β, IL-1ra, IL-6, IL-8 and IL-10), which have previously been shown to respond to acute exercise and play a role in regulating muscular regeneration (Peake et al. 2015; Pedersen and Hoffman-Goetz 2000), in response to acute strength exercise between naturally cycling women and women using oral contraceptives. To enable comparison between these groups, women were either tested in the early follicular phase of the menstrual cycle, where oestradiol and progesterone concentrations are generally low, or in the pill-free interval, where there is no acute intake of exogenous hormones.

## Methods

### Experimental design

**Fig. 1:**
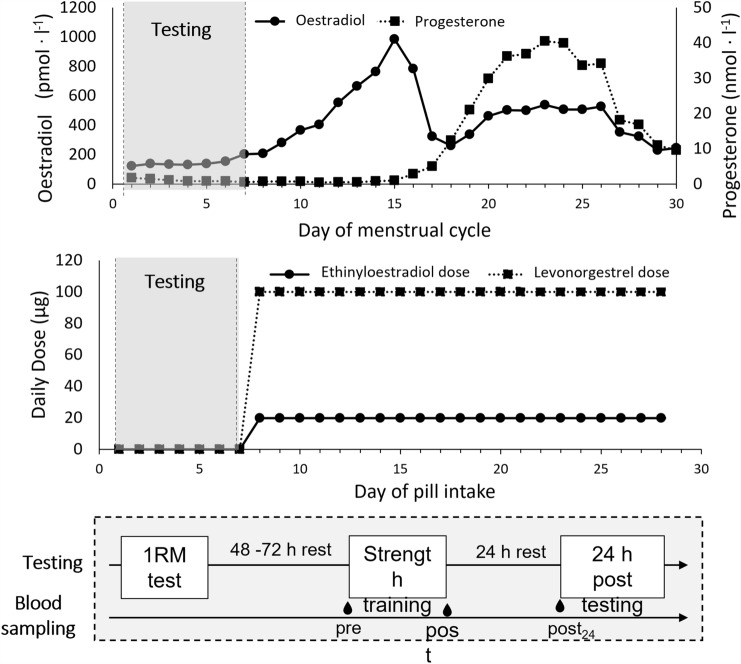
Timing of testing in the natural menstrual cycle and oral contraceptive intake (exemplary data modified from Sims and Heather (2018). Testing was performed in the early follicular phase or pill-free interval of the respective cycle. 1RM = one-repetition maximum in the back squat

The study is a secondary analysis of a previous study investigating the effects of acute strength exercise in naturally cycling women (MC) and oral contraceptive users (OC) on steroid hormones and the tryptophan metabolism (Umlauff et al. 2021). The study consisted of a maximal strength test and a strength training session in the low-hormone phase in the first days of the respective cycle, i.e., in the early follicular phase of the MC group and the pill-free interval of the OC group (see Fig. 1). The menstrual cycle phase was determined by the onset of menstrual bleeding. Firstly, the one-repetition maximum (1RM) of participants in the deep back squat was determined with a maximal strength test. This test was performed on days 1–3 after the onset of menstruation (MC) or the withdrawal bleed (OC). After 48 to 72 h recovery, a strength training session was performed. This was performed between days 3–6 of the respective cycle. The strength training session consisted of a warm-up on a cycle ergometer and 4 × 10 repetitions at 70% 1RM in the deep back squat. Blood sampling and explosive strength testing was performed before (pre), directly after (post) and 24 h after the training session (post_24_). Participants arrived in a fasted state and rested on a chair for 5 min before the first blood sampling. They then proceeded to have a light breakfast at least 15 min prior to starting the warm-up. Similarly, participants also arrived in a fasted stated for the post_24_ testing. Testing was performed at similar times of the day (± 1 h) and participants were instructed to refrain from alcohol and any intense physical activity at least 24 h prior to testing.

### Participants

Twenty-four healthy women with strength training experience, defined by 1RM in the back-squat between 0.8 and 1.5-times body mass and at least two strength training sessions per week for a minimum of two years, originally participated in the study. Three women using contraceptives had to be excluded due to timing of testing, insufficient maximal strength (1RM < 0.8 × body mass) and abnormal cortisol levels, therefore, 21 women were included in the final analysis. Women belonged to either one of two groups: (1) a group (MC; *n* = 13, age: 24 ± 4 years, weekly strength training: 4.3 ± 1.7 h) with a regular natural menstrual cycle (± 2 days) and no use of oral contraceptives (last use > 8 months) or (2) a contraceptive group (OC; *n* = 8, age: 22 ± 3 years, weekly strength training: 4.5 ± 1.9 h) with the regular use (> 9 months) of a monophasic combined pill (21 + 7 intake scheme with 30 µg ethinyloestradiol + 20 µg chlormadinone or dienogest). Smokers, women experiencing menstrual cycle irregularities (> ± 2 days) or amenorrhea in the last 12 months or women using emergency contraceptives or other hormone-affecting medication in the last 3 months were excluded from participating in this study. Before commencing with the study, all participants were informed of possible risks and provided written consent. The study was approved by the German Sport University Institutional Review Board (020/2019) and also conducted in accordance with the Declaration of Helsinki and its later amendments. Recruitment and testing were performed by different study personnel, therefore the person performing data analysis was blinded.

### Procedures

*Maximal strength testing* To determine the 1RM of participants in the back squat, a maximal strength test was performed using a Smith Machine (Gym80 international GmbH, Gelsenkirchen, Germany). For the warm-up, 5 min cycling on a cycle ergometer (1.5 W kg^−1^), unweighted squat exercise and six repetitions with a guided 22 kg bar were performed. Thereafter, the 1RM was determined using a force–velocity based approach. Participants were asked to descend their hips below their knees and rest periods between sets were standardised to 2 min. A more detailed description of testing and participant characteristics can be found in Umlauff et al. 2021.

*Explosive strength testing* Indices of neuromuscular fatigue were assessed by using an explosive strength protocol, which consisted of three repetitions at 60% 1RM. Participants were instructed to perform a slow and controlled eccentric phase and the following concentric phase explosively and as fast as possible. Mean propulsive velocity (MPV) was recorded for each repetition using T-FORCE Dynamic Measurement System (ERGOTECH Consulting, S.L., Pamplona, Spain) and averaged for each sampling timepoint.

*Strength training session.* Between day 3 and 6 of the cycle, a strength training session was performed using a Smith Machine. A similar warm-up to maximal strength testing was performed, which was followed by 4 × 10 repetitions at 70% 1RM in the back squat with 2 min rest between sets. This protocol was based on a protocol (3 × 12 repetitions at 70% 1RM), which had previously been shown to produce a marked physiological response in men (Pareja-Blanco et al. 2017). As women show increased time to task failure and faster neuromuscular recovery, we modified the protocol to include 4 sets and in pilot testing found this to be sufficient (Hunter 2009). Participants performed the exercise with constant velocity in both concentric and eccentric phases and a two-second pause between each repetition. Study personnel provided assistance, if participants were unable to perform the concentric phase of the squat, so that all participants had an identical number of repetitions.

*Blood sampling and analysis* Blood samples were collected from the antecubital vein into heparin-coated serum containers, for pre and post_24_ after 5 min of rest in a sitting position and post directly after testing ended. After clotting for 10 min, samples were centrifuged at 1900 g for 10 min. Serum was aliquoted and stored at -80 °C for further analysis. Cytokine concentrations were measured by enzyme-linked immunosorbent assay (ELISA) for IL-1β (*IL-1beta Human ELISA Kit, BMS224-2*, *Invitrogen, ThermoFisher Scientific, Massachusetts, USA)*, IL-1ra (*IL1RA Human ELISA Kit, KAC1181*, *Invitrogen, ThermoFisher Scientific, Massachusetts, USA)*, IL-6 (*IL-6 High Sensitivity Human ELISA Kit, BMS213HS, Invitrogen, ThermoFisher Scientific, Massachusetts, USA*), IL-8 (*High Sensitive ELISA Kit for IL-8, HEA080Hu, Cloud Clone Corp., Katy, USA)* and IL-10 (*IL-10 High Sensitivity Human ELISA Kit, BMS215HS, Invitrogen, ThermoFisher Scientific, Massachusetts, USA*). Samples were analysed in duplicate. The intra-assay CV were as follows: IL-1β—5.0%, IL-1ra—6.5%, IL-6—6.2%, IL-8—8.8%, IL-10—6.4%. One data point for IL-1β and two data points for IL-6 were outside the sensitivity limits, so the corresponding participants were not included in the analysis for the respective cytokines. Oestradiol and cortisol were analysed previously (Umlauff et al. 2021).

### Statistical analysis

An a-priori power analysis was performed for the original study using G*Power 3 with an expected medium effect size of 0.3, alpha = 0.5 and power = 0.8 and projected a participant number of *n* = 20 (Umlauff et al. 2021). Data were analysed using SPSS version 29.0 (SPSS, IBM Statistics, New York, US). Firstly, normality and homoscedasticity were checked by using the Shapiro–Wilk Test and visually inspecting residual histograms, residual plots and Q-Q plots. A log-transformation was used to achieve normality for non-normally distributed data (IL-1β, IL-6). Baseline differences were analysed by using a two-tailed independent t-tests. For time and interaction effects, a mixed repeated measure analysis of variance (ANOVA) was performed with Bonferroni correction for post-hoc tests. Measurement timepoints for the cytokines were defined as within-group variables (i.e., pre, post and post_24_) and the group (OC, MC) was defined as the between-group variable. Effect sizes for main effects of the ANOVA are reported as partial η^2^ and for the comparison between time-points Hedges g was calculated. To assess associations between the parameters (oestradiol, cortisol, IL-1β, IL-1ra, IL-6, IL-8, IL-10 and MPV) across all conditions Spearman’s rank correlation coefficient was calculated. For all tests, statistical significance was accepted at *p* ≤ 0.05. All data are presented as mean ± standard deviation (SD). For absolute concentrations of oestradiol, cortisol and cytokines 95% confidence intervals (CI) were also calculated.

## Results

### Participant characteristics

Participants did not differ in age, height, weight, BMI, 1RM or training experience (Table 1, all p > 0.05).

**Table 1:** Characteristics of the menstrual cycle (MC) and oral contraceptive (OC) group

	MC	OC
Age (yrs)	24 ± 4	22 ± 3
BMI (kg m^−2^)	22.4 ± 2.6	21.5 ± 1.8
1RM (kg kg bodyweight^−1^)	1.1 ± 0.2	1.1 ± 0.1
Weekly strength training (h week^−1^)	4.3 ± 1.7	4.5 ± 1.9
Day of cycle tested	5 ± 0.7 (4—6)	4 ± 0.8 (3—5)

Data are presented as mean ± SD. For day of cycle tested, the range is also presented.

### Cytokines

**Fig. 2:**
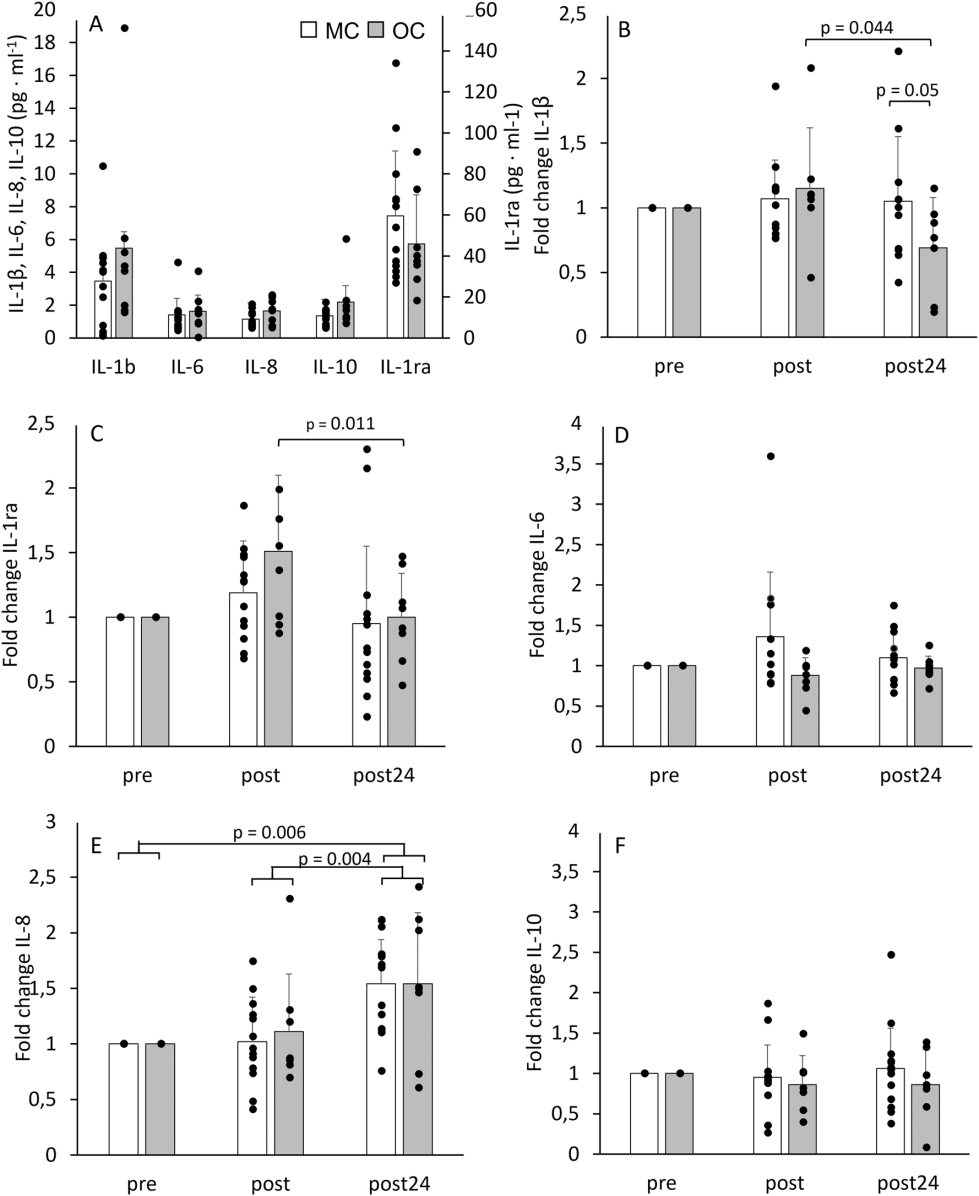
Baseline concentrations at pre of all cytokines (**A**) and fold change from baseline values for IL-1β (**B**), IL-1ra (**C**), IL-6 (D), IL-8 (**E**) and IL-10 (**F**). For IL-1β analysis was performed for *n* = 20 (MC: 13, OC: 7) and for IL-6 *n* = 19 (MC:11, OC: 8). Data are shown as mean ± SD, with black dots showing individual values

Concentrations of cytokines throughout the strength training sessions are presented in Table 2. There were no baseline differences for all cytokines between the MC and OC group (p > 0.05). For IL-1ra, there was a main effect for time in the OC group only (*p* = 0.027, *η*^2^ = 0.174). In the OC group, concentrations of IL-1ra increased by + 51.1 ± 59.4% from pre to post (*p* = 0.189, *g* = 0.693) and statistically decreased by -20.5 ± 13.5% from post to post_24_ (p = 0.011, *g* = 1.348) (Fig. 2C). There were no statistical changes in the MC group (all p > 0.05) and there was no main interaction effect (*p* = 0.360, *η*^2^ = 0.052). For IL-1β, there was no main effect for time (*p* = 0.216, *η*^2^ = 0.084), however there was an interaction effect (*p* = 0.038, *η*^2^ = 0.200). At post_24_ concentrations of IL-1β were statistically lower in the OC group compared to MC (*p* = 0.05, *η*^2^ = 0.193). The OC group showed a main effect for time (*p* = 0.033, *η*^2^ = 0.435), with concentrations of IL-1β not statistically changed from pre to post (*p* = 1.000) but statistically decreased by – 39.6 ± 23.0% from post to post_24_ (*p* = 0.044, *g* = 0.971) (Fig. 2B). There was no main effect for IL-6 for time (*p* = 0.599, *η*^2^ = 0.030) or group (*p* = 0.723, *η*^2^ = 0.019) (Fig. 2D). Similarly, for IL-10 there was no main effect for time (*p* = 0.489, *η*^2^ = 0.039) or group (*p* = 0.269, *η*^2^ = 0.070) (Fig. 2F). For IL-8, there was a main effect for time across both groups (*p* < 0.001, *η*^2^ = 0.358) but no interaction effect (p = 0.921, η^2^ = 0.004). Concentrations of IL-8 statistically increased by + 66.6 ± 96.3% from post to post_24_ across both groups (*p* = 0.004).

**Table 2:** Concentrations of cytokines, oestradiol and cortisol

	MC			OC		
Variable	Pre	Post	Post_24_	Pre	Post	Post_24_
Oestradiol (pg ml^−1^)	29.7 ± 13.6 [21.4; 37.9]	23.8 ± 9.1 [18.4; 29.3]	28.6 ± 19.6 [16.8; 40.5]	18.7 ± 12.7 [8.1; 29.4]	15.7 ± 8.7 [8.4; 22.9]	17.3 ± 7.8 [10.8;23.8]
Cortisol (ng ml^−1^)	248.6 ± 62.1^†^ [211.1; 286.2]	205.8 ± 66.4^#^ [165.5; 245.8]	210.4 ± 72.1 [166.9; 254.0]	490.4 ± 80.7^†^ [422.9; 557,9]	435.7 ± 92.4^#^ [358.4; 513.0]	451.6 ± 81.8 [383.3; 520.0]
IL-1β (pg ml^−1^)	3.5 ± 2.8 [1.8; 5.2]	3.5 ± 2.6 [2.0; 5.1]	3.5 ± 2.2^†^ [2.2; 4.8]	3.6 ± 1.8 [1.9; 5.3]	4.0 ± 2.7 [1.5; 6.5]	2.6 ± 1.9*^, †^ [0.8; 4.3]
IL-1ra (pg ml^−1^)	59.6 ± 31.6 [40.4; 78.7]	63.0 ± 20.2 [50.0; 75.2]	52.3 ± 39.0 [28.7; 75.9]	46.0 ± 23.9 [26.0; 66.0]	65.9 ± 35.4 [36.3; 95.5]	45.3 ± 27.8* [22.1; 68.6]
IL-6 (pg ml^−1^)	1.4 ± 1.1 [0.6; 2.2]	1.9 ± 1.7 [0.7; 3.0]	1.3 ± 0.7 [0.9; 1.8]	1.6 ± 1.2 [0.6; 2.6]	1.4 ± 1.0 [0.6; 2.2]	1.5 ± 1.1 [0.6; 2.5]
IL-8 (pg ml^−1^)	1.1 ± 0.5 [0.8; 1.4]	1.1 ± 0.4 [0.8; 1.3]	1.7 ± 0.9* [1.2; 2.3]	1.6 ± 0.8 [1.0; 2.3]	1.6 ± 0.6 [1.1; 2.1]	2.2 ± 0.9 [1.4; 3.0]
IL-10 (pg ml^−1^)	1.4 ± 0.5 [1.1; 1.6]	1.3 ± 0.6 [1.1; 1.7]	1.5 ± 0.8 [0.9; 1.9]	2.2 ± 1.8 [0.6; 3.8]	2.0 ± 0.1.9 [0.2; 3.7]	1.8 ± 1.6 [0.3; 3.3]

Data are given as mean ± SD [95% Confidence Interval]

*Statistical change from post to post_24_

^#^Statistical change from pre to post

^†^Statistical difference between groups

### Explosive strength, oestradiol and cortisol

Detailed results for these parameters have previously been published (Umlauff et al. 2021). Briefly, there were no statistical baseline differences between groups for oestradiol (*p* = 0.083) and MPV (*p* = 0.068). Cortisol concentrations at baseline were statistically increased in the OC compared to the MC group (*p* < 0.001, *η*^2^ = 0.759). Oestradiol concentrations are in line with the early follicular phase of the MC group. MPV and oestradiol did not statistically change throughout the intervention (oestradiol: *p* = 0.307, *η*^2^ = 0.060, MPV: *p* = 0.075, *η*^2^ = 0.127) and there were no between group differences. Cortisol showed a main effect for time (*p* = 0.002, *η*^2^ = 0.325) but no interaction effect (*p* = 0.570, *η*^2^ = 0.024).

### Pooled associations between cytokines, oestradiol, cortisol and explosive strength

There were no associations between cytokine concentrations and 1RM, oestradiol, and cortisol concentrations (*p* > 0.05). However, there were some associations between single cytokines. At post and post_24_ there was an association between concentrations of IL-10 and IL-6 (*r* = 0.487, *p* = 0.004, *r* = 0.651, *p* = 0.003, respectively) across all subjects. Additionally, at post_24_, changes in IL-10 were associated with changes in IL-1β (*r* = 0.472, *p* = 0.041) and IL-1ra (*r* = 0.514, *p* = 0.02) and changes in IL-8 were associated with changes in IL-1ra (*r* = – 0.495, *p* = 0.018).

## Discussion

The aim of this study was to compare the response of cytokines to acute strength exercise between naturally cycling women in the early follicular phase and oral contraceptive users in the pill-free interval. At baseline, we found no differences between these groups in any of the cytokines. In response to strength exercise, IL-6 and IL-10 remained unaltered in the two groups. For IL-8, similar responses were found across both groups, with concentrations increasing from post to post_24_. On the other hand, IL-1β and IL-1ra showed differential regulation between groups, with concentrations of IL-1β and IL-1ra decreasing only in the OC group from post to post_24_. IL-1β also showed a statistical between group difference, with concentrations differing statistically at post_24_.

When looking at baseline differences of cytokines, results from previous studies have been conflicting. Some studies have proposed a reduction of inflammatory status with OC use, for example through reductions of neutrophils and IL-8 (Giraldo et al. 2008), while others have found an increased inflammatory status with higher concentrations of CRP and TNF-α (Cauci et al. 2017; Rickenlund et al. 2005). However, comparison between studies is difficult, as pill and cycle phases are often not described or accounted for. Nevertheless, some well-controlled studies exist. In contrast to our results, two studies found IL-6 concentrations to be decreased in the pill-free interval compared to the early follicular phase (Eagan et al. 2021; Souter et al. 2005), while we found no difference between the pill-free interval and the early follicular phase in this study. Participants in these studies were described only as healthy, whereas participants in this current study were resistance trained and participating in regular physical activity. As regular exercise promotes an anti-inflammatory environment, this may be an explanation for the lack of difference between the two groups in our study. Similarly, Larsen et al. (2020) found no difference in any cytokines, including IL-6, between naturally cycling elite athletes and those using oral contraceptives, although menstrual cycle phases were mixed (Larsen et al. 2020). For the other cytokines, there are less well controlled studies comparing baseline concentrations between OC users and naturally cycling women. Giraldo et al. (2008) showed decreased IL-8 concentrations in OC users compared to naturally cycling women (Giraldo et al. 2008), while others found no difference (Larsen et al. 2018; Welsh et al. 2008). For IL-1β, IL-1ra, and IL-10, studies have shown no baseline differences, which is in line with our results (Cullup et al. 2004; Michel et al. 2015; Sikora et al. 2015).

Furthermore, the lack of information regarding the composition of the oral contraceptive used also limits interpretation of previous results, as the spectrum of synthetic progestins vary in their effects regarding estrogenic, androgenic and progestogenic activity, possibly masking unambiguous results. Although the effect of different progestins in oral contraceptives has not yet been investigated on cytokines, studies have for example shown differing effects on adipokines (Di Carlo et al. 2011; El-Haggar and Mostafa 2015) and natural killer cells (Auerbach et al. 2002), dependent on the composition of the oral contraceptive. Therefore, different synthetic progestins could influence the release of cytokines differently. However, the oral contraceptives used in the present study were limited to formulations containing chlormadinone acetate or dienogest as the progestin component only. Both have strong antiandrogenic and low to no estrogenic activity (Bouchard 2005; Prez-Campos 2010), therefore allowing for a clearer interpretation of the results.

Changes in cytokines in response to the strength exercise were mainly seen 24 h after exercise, in the recovery period. However, only certain cytokines were regulated and a difference in regulation between OC users and naturally cycling women was found for IL-1β and IL-1ra only. In contrast, after a three-stage cycling trial (total exercise duration: 52.5 min), no statistical change was detected in IL-1β and IL-1ra concentrations and no difference was found between OC users and naturally cycling women (Larsen et al. 2018). However, blood was only sampled directly post exercise in the aforementioned study, while in this current study changes were mainly found 24-h post exercise. More specifically, in this study, while not statistically significant but showing an intermediate effect (*g* = 0.693), OC users showed an increase in IL-1ra directly after strength exercise (post: + 51 ± 59%) and also showed a statistical decrease at post_24_ with a concurrent decrease in IL-1β. These two cytokines are antagonistic in their activity, with IL-1β being released in response to muscle damage to facilitate a pro-inflammatory response for muscle repair. IL-1ra has anti-inflammatory activity and can decrease IL-1β activity (J. E. Sims and Smith 2010). Therefore, it could be hypothesised that the antagonistic properties of IL-1ra are responsible for the significant decrease in IL-1β in OC users at post_24_. However, statistical associations were not found between IL-1ra and IL-1β (i.e., no significant correlations at any timepoints or in any group) and therefore are not able to support this interaction.

Regarding endogenous oestradiol concentrations, no differences were found between groups and concentrations were low, which is in line with previous findings. However, when looking at the groups separately, MC participants in the early follicular phase are experiencing the lowest hormone concentrations of the cycle. In contrast, it is known that in OC users, endogenous concentrations of oestradiol increase slightly throughout the pill-free interval, as exogenous hormones are not suppressing the release of endogenous oestrogen in the pill-free interval. Therefore, while objectively endogenous concentrations were similar, OC users and MC users are experiencing the highest and lowest concentrations of their cycle, respectively. This could partly explain the differing response between the two groups in IL-1β and IL-1ra. Indeed, oestrogen has been shown to reduce the mRNA synthesis and secretion of IL-1β and IL-1ra in monocytes in in vitro experiments (Morishita et al. 1999; Polan et al. 1989) and also postmenopausal women treated with oestrogen replacement therapy show a reduction in IL-1β and IL-1ra (Pacifici et al. 1993). Furthermore, high oestrogen and progesterone concentrations have also been found to suppress the release of IL-6 and IL-1ß, while low oestrogens concentrations can stimulate this release (Klein and Flanagan 2016; Straub 2007). Therefore, there may be a differing dependency on endogenous oestrogen concentrations as well as chronic oestrogen exposure between oral contraceptive users and naturally cycling women (Campesi et al. 2012). This could be an explanation for the decrease of IL-1β and IL-1ra after 24 h in response to exercise in the OC group only, but it is speculative and further investigations are necessary. Similarly, changes in oestrogen receptor sensitivity or binding proteins should be investigated. For example it is known that oral contraceptive users show increased concentrations of sex-hormone binding globulin (Wiegratz et al. 2003), which has been shown to have an inverse relationship with pro-inflammatory cytokines and anti-inflammatory effects on adipocytes and macrophages (Maggio et al. 2011; Yamazaki et al. 2018). However, to assess this was beyond the scope of this study.

Interestingly, both groups showed a similar response to the strength exercise in IL-8, which increased by + 67 ± 96% 24 h after the strength exercise. IL-8 can be transiently released in small concentrations by working muscles, however, in response to exercise, it is mainly released due to a systemic release of pro-inflammatory cytokines (Hoffmann et al. 2002). IL-8 shows inflammatory and chemokine properties in attracting neutrophils to sites of muscle damage (Pedersen et al. 2007). However, IL-8 has previously been found to be released after extensive endurance exercise or eccentric exercise and not commonly after strength exercise (Chan et al. 2004; Henson et al. 2000), where muscle damage is more likely to occur. We did not assess any markers of muscle damage, however, the testing protocol did not induce reductions in explosive strength performance (i.e., a surrogate of neuromuscular fatigue). Nevertheless, as both groups show a similar increase, it can be postulated that this response to exercise is not modulated by oral contraceptive use, when compared to the early follicular phase.

The lack of reductions in explosive strength performance suggest that the protocol did not induce a measurable level of neuromuscular fatigue, unlike it was previously shown to in men (Pareja-Blanco et al. 2017). In line with this, women have been shown to have an increased time to task failure, as well as faster recovery of neuromuscular function compared to men (Hunter 2009). Therefore, it can be speculated that for certain cytokine responses the volume of the strength protocol was possibly not high enough to induce a marked response. This may somewhat explain, why we did not see any responses or between group differences in IL-6, which shows a stronger released with increasing intensity but especially with increasing duration of exercise (Pedersen and Febbraio 2008). This is also in contrast to a study by Timmons et al. (2005) showing a lower IL-6 response in the pill-free interval of oral contraceptive users compared to the follicular phase of the menstrual cycle (Timmons et al. 2005). However, it has to be mentioned, that exercise mode was very different (90 min continuous cycling at 65% VO_2_max), which makes a direct comparison difficult. Nevertheless, we found exercise-induced responses in some cytokines (IL-1β, IL-1ra and IL-8) and differences in responses between groups. Therefore, the exercise protocol induced an inflammatory response despite the lack of reductions in explosive strength. Whether a more intense and longer exercise protocol may induce a stronger inflammatory response and greater differences between groups cannot be ultimately concluded and remains to be investigated.

Importantly, in this study, we only assessed the acute response to strength exercise and therefore, the consequences, if any, of this differential regulation of IL-1β and IL-1ra between naturally cycling women and oral contraceptive users on neuromuscular adaptations, such as hypertrophy need to be further investigated. For example, Hansen et al. (2011) found a lower myofibrillar synthesis rate in OC users with a low androgenic pill compared to naturally cycling women (Hansen et al. 2011), while Dalgaard et al. found a trend (p = 0.06) towards a larger increase in muscle mass in OC users with similar low androgenic pills (Dalgaard et al. 2019). Ihalainen et al. (2019) showed higher concentrations of hs-CRP in hormonal contraceptive users after 10 weeks of combined strength and endurance training, along with smaller gains in lean body mass compared to naturally cycling women, suggesting that inflammation may be linked to smaller muscular adaptations in response to training (Ihalainen et al. 2019). Therefore, results have been conflicting and the involvement of inflammatory parameters in muscle adaptation in longitudinal training studies in this setting largely remains to be investigated.

In conclusion, IL-1β and IL-1ra showed a differential regulation in response to acute strength exercise between oral contraceptive users and naturally cycling women, with concentrations of both cytokines decreasing 24 h post exercise in the OC group only. IL-6 and IL-10 did not change over time and also showed no between group differences, while IL-8 increased across both groups 24 h post exercise. Therefore, it is proposed that the long-term use of oral contraceptives chronically affect the response of certain cytokines, like IL-1β and IL-1ra, even when testing in the pill-free interval of oral contraceptive users. This disparate regulation highlights the fact that research should not be investigating inflammatory status per se but instead view inflammatory processes differentially. It remains to be further analysed, how these inflammatory responses change throughout the full menstrual cycle, when variations in hormonal concentrations increase. Additionally, the exact physiology behind these differences and long-term implications for adaptations to strength exercise remain to be investigated.

## Data Availability

The data generated and analysed during the current study are available from the corresponding author on reasonable request.

## Abbreviations

1RM: One-repetition maximum
IL: Interleukin
IL-1ra: Interleukin-1 receptor antagonist
OC: Oral contraceptive
MC: Menstrual cycle
Th1/2: T helper type 1/2 response
TNF-α: Tumour necrosis factor alpha
